# Having more friends is associated with greater sensitization to social exclusion: neural and behavioral evidence

**DOI:** 10.1093/scan/nsaf067

**Published:** 2025-06-30

**Authors:** Elisa C Baek, Yixuan Lisa Shen, Hairin Kim, Ekaterina Baldina, Jeanyung Chey, Yoosik Youm, Carolyn Parkinson

**Affiliations:** Department of Psychology, University of Southern California, Los Angeles, California 90089, United States; Department of Psychology, University of California, Los Angeles, Los Angeles, California 90095, United States; Department of Psychology, Seoul National University, Seoul 08826, South Korea; Department of Sociology, Indiana University Bloomington, Bloomington, Indiana 47405, United States; Department of Psychology, Seoul National University, Seoul 08826, South Korea; Department of Sociology, Yonsei University, Seoul 03772, South Korea; Department of Psychology, University of California, Los Angeles, Los Angeles, California 90095, United States; Brain Research Institute, University of California, Los Angeles, Los Angeles, California 90095, United States

**Keywords:** social network analysis, social rejection, loneliness, fMRI

## Abstract

Social rejection profoundly affects well-being. How do features of people’s real-world social networks relate to responses to social exclusion? People central in social networks—e.g. who have many friends—may be less distressed by exclusion, since they have many sources of support, or more sensitive to it, if they are more attuned to social feedback. We characterized a village’s social network; a subset of residents (*N *= 74) completed a functional magnetic resonance imaging (fMRI) study involving social exclusion in groups characterized by varying social relationships (spouses, friends, strangers). Highly-central individuals reported greater distress during exclusion by strangers, and their distress ratings scaled with responses in brain regions associated with social pain, negative affect, and mentalizing. Thus, while social connectedness is often considered a protective factor that promotes well-being, these findings suggest a potential ironic effect of social connectedness–vulnerability to distress in particular social contexts. This sensitization to exclusion could constitute an adaptive tendency to promote long-term wellness.

## Introduction

Humans are inherently social, with fundamental needs for social connection (Baumeister and Leary 1995). Social relationships are consequential in determining individuals’ well-being, with deficits in social connection linked to detrimental effects, including increased risk of mortality (House et al. 1982, 1988, Uchino 2006, Youm et al. 2021). Accordingly, experiencing negative interpersonal interactions, such as exclusion, can cause significant distress; indeed, neuroimaging evidence suggests that social exclusion is associated with brain activity that is consistent with negative affect and emotional pain (Eisenberger et al. 2003, Wang et al. 2017).

However, little is known about how features of an individual’s social world (e.g. how well-connected an individual is) along with the context of the exclusion in their social environment (e.g. who is the source of the exclusion) are linked to their behavioral and neural responses to social exclusion. This is partly because most studies investigating social exclusion have done so in contexts where participants are excluded by strangers, or anonymous virtual others, without considering features of the participants’ real-world social networks (Wirth 2016). For instance, as described in more detail below, individuals who are central in their social network (e.g. who have many friends) may be less distressed by exclusion, if the existence of strong social support buffers against negative interpersonal experiences. On the other hand, highly-central individuals may be more sensitive to exclusion, if they are more attuned to social feedback, either as a cause or consequence of interacting with many social partners. Here, we used neuroimaging, self-report, and social network data to arbitrate between these competing hypotheses and test the association between social network centrality and sensitivity to social exclusion.

Individuals who are central in their social networks and interact with many social partners generally experience higher well-being and less loneliness compared to individuals who are less well-connected, due to the greater support that they experience from having many social ties (Russell et al. 1984, House et al. 1988, Shiovitz-Ezra and Leitsch 2010). Accordingly, one possibility is that people who have many friends are *less* affected by exclusion because their many social connections serve as protective mechanisms against the negative consequences of exclusion. Corroborating this theory, in children and adolescents, both the number and quality of social ties attenuate behavioral and neural sensitivity to social rejection (Bagwell et al. 2001, Masten et al. 2012). This small body of literature that supports the buffer hypothesis has focused on developmental samples, during periods of life marked by tremendous change (Blumenthal et al. 1999). Here, we tested whether being well-connected is associated with more or less attunement to social rejection among adults in later life stages.

An alternative hypothesis is that central individuals may be *more* sensitive to exclusion since they are more attuned to recognizing and incorporating social feedback. The capacity to accurately understand others’ mental states, behaviors, and motivations is important for maintaining healthy psychosocial functioning (Hall et al. 2009), and one theory posits that social pain shares a similar pathway with physical pain, serving as a ‘neural alarm system’ to protect us from social and physical threats (Eisenberger and Lieberman 2004); as such, increased sensitivity to negative social feedback may be one adaptive behavior that distinguishes highly-central individuals. Indeed, people who have greater desires to connect with others (i.e. greater need to belong) show heightened sensitivity to social cues (Pickett et al. 2004), and individuals who connect otherwise unconnected others show increased neural reactivity in regions of the brain associated with social cognition when told that their preferences are misaligned with those of their peers (O’Donnell et al. 2017). Thus, one possibility is that highly-­central individuals are more sensitive to negative social experiences such as social exclusion.

Here, we conducted a secondary data analysis on a larger dataset (i.e. The Korean Social Life, Health, and Aging Project (KSHAP); Baek et al. 2024) to specifically use social network analysis and neuroimaging to arbitrate between these competing hypotheses. We characterized the social network of a village and computed each participant’s social network centrality based on the number of people with whom they reported regularly discussing important matters (i.e. out-degree centrality). Since we aim to test for links between subjective feelings of distress during social exclusion and one’s perceived level of social connectedness, we focused on out-degree centrality as our social network-based variable of interest as it captures one’s perceived number of social ties. A subset of villagers attended a neuroimaging session where their brain activity was measured while they played Cyberball, a virtual ball-tossing game that has been validated to elicit feelings of social exclusion (Williams et al. 2000, Hawkley et al. 2011, Wang et al. 2017). Participants attended the sessions in groups of three, and during Cyberball, each participant believed that they were excluded by the other two participants with whom they attended the session. Based on prior work (Eisenberger et al. 2003, Masten et al. 2011, Beyer et al. 2014, Wang et al. 2017), we extracted mean activity in three brain systems—social pain, negative affect, and mentalizing—during social exclusion, and linked that activity with distress during exclusion. This approach allowed us to test whether individuals who have more friends show greater or less neural and behavioral sensitivity to exclusion. Further, we leveraged the natural variation in the existing relationships between participants (e.g. some participants attended the study session with their friend or spouse, whereas others attended with strangers) to explore whether participants’ relationships with their Cyberball partners affected responses to exclusion.

Our findings provide support for the hypothesis that highly-central individuals exhibit greater sensitivity to social exclusion. People with many friends showed greater neural sensitivity to social exclusion and reported greater distress during exclusion by strangers, although they reported less overall subjective social disconnection (i.e. loneliness) in everyday life. Our findings provide insight into how features of individuals’ real-world social networks—such as number of friends—may be related to how they respond to negative social experiences.

## Materials and methods

### Participants

Participants were recruited as part of a longitudinal project (i.e. KSHAP; Baek et al. 2024) that studies various health determinants of older adults living in rural areas in South Korea. We collected data from Township L, a traditional rural village located in the east of the Ganghwa island with an area of 31.45 km^2^ (Baek et al. 2024). By studying relationships between social network centrality and sensitivity to social exclusion in a non-WEIRD sample (Arnett 2008, Dan 2010), we seek to move towards an enriched understanding of the psychological and neural processes involved in social exclusion.

#### Social network and loneliness data

According to the 2015 Resident Registration data of Township L, 1044 residents were eligible to participate in the study. A total of 948 residents (90.8% response rate) completed the social network survey, which included questions about their social relationships and their physical and mental health (see below for further details).

#### Neuroimaging data

A total of 110 participants from Township L took part in the neuroimaging portion of the study. Given that the current investigation is part of a large longitudinal study of older adults, we sought to maximize the number of subjects that could be successfully recruited from the Township; our sample size of 110 reflects this effort. Although our sample size was not predetermined by power analysis based on effect size estimates, a *post-hoc* power analysis suggests that we had 0.83 power to detect an effect size of *f^2^* = 0.079 (the smallest effect size we observed in our main analysis predicting subjective emotional distress from the interaction of brain activity and out-degree centrality; see Methods and Results for more details) with an *N* of 110, and 0.66 power with an *N* of 74 (our final *N* due to exclusions; see below). Fourteen participants were excluded because they did not believe the Cyberball cover story or did not recognize that they were being excluded during the exclusion round of the ball-tossing game. Six participants were excluded due to the detection of brain disease. Six additional participants were excluded due to technical problems and missing data. Fourteen participants were excluded due to excessive head movement (as defined by greater than 10% of the frames showing framewise displacement of 0.5 mm or greater). Two additional participants did not complete the post-scan questionnaires. As a result, data from 74 participants (M_age_ = 73.07, SD_age_= 6.49; 42 female) were included for analyses. All participants gave informed consent in accordance with the procedures of the Institutional Review Boards of the Yonsei University and Yonsei University Health System, Severance Hospital, where the study took place.

### Procedure

#### Social network and loneliness data

The social network questionnaire was administered in the participants’ homes and community centres (between December 2016 and March 2017). As a part of this questionnaire, participants were asked to indicate up to six people with whom they most often discussed important issues within the past 12 months (‘From time to time, most people discuss things that are important to them with others. For example, good or bad things that happen to you, problems that you are having, or important concerns that you may have. Looking back over the last 12 months, who are the people with whom you most often discussed things that were important to you?’) which was adapted from prior work (Burt 1984, Cornwell et al. 2009, Youm et al. 2014). The cap of six nominations was imposed on the social network survey to help alleviate participants’ burden and retain their attention and is consistent with other studies of large social networks (e.g. Burt 1984). This cap was chosen given that the name generator used was focused on eliciting the names of participants’ close friends (e.g. those with whom they would likely discuss important matters). Whereas people tend to have 12–15 regular social contacts, most people tend to have approximately 4–5 close friends (sometimes referred to as their ‘support clique;’ Dunbar and Spoors 1995, Roberts et al. 2008) and 2–3 people with whom they discuss important matters (McPherson et al. 2006, Brashears 2011, Hampton et al. 2011). We used participants’ responses to this name generator to calculate out-degree centrality, with a potential minimum of zero and a maximum of six.

Participants also answered questions about their mental and physical health, including the Center for Epidemiological Studies Depression Scale (CES-D) (Lewinsohn et al. 1997), which was translated and validated in Korean (Cho and Kim 1998; ‘Below is a list of the ways you might have felt or behaved. Please tell me how often you have felt this way during the past week’). Participants’ answers to the fourteenth item of the CES-D (‘I felt lonely’ – rarely or none of the time (less than 1 day), some or a little of the time (1–2 days), occasionally or a moderate amount of time (3–4 days), most or all of the time (5–7 days)) were used to quantify overall loneliness. Validating our approach, this single-item measure of loneliness is correlated with other multi-item scales for loneliness (Hughes et al. 2004, Hanratty et al. 2018) and has been used in prior work to relate loneliness to behavioral outcomes (Fokkema et al. 2012, Menec et al. 2019).

#### Neuroimaging appointment

Participants attended the neuroimaging appointment in groups of three (i.e. an individual attended the study appointment with two other individuals who were living in the same village). Prior to entering the MRI scanner, participants filled out a pre-scan questionnaire, which included questions about participants’ relationships with the other two participants who attended the same study appointment. To minimize potential discrepancies due to inaccurate memory, participants were asked whether they knew one another, and, if so, the number of years that they had known each other, in a setting where all participants were present. Then, participants were moved to a private room and were asked to indicate the categorical nature of their relationship with each of the two participants (i.e. ‘What is the nature of your relationship with participant X?’) out of five potential categories (i.e. stranger, friend, neighbour, family member, and spouse).

**Figure 1.**
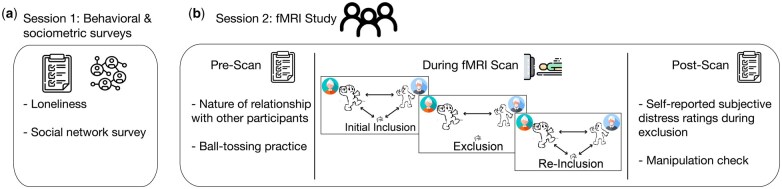
Illustration of the study procedure. (a) Session 1 took place in participants’ homes and community centres and consisted of questionnaires that included self-report measures of loneliness and information about participants’ social relationships. (b) Participants attended the fMRI study in groups of three. Prior to entering the scanner, participants completed questionnaires that included information about the nature of their relationships with the other two participants who attended the study session with them. They also practiced playing the ball-tossing game that they would play in the scanner by throwing a physical ball to one another. During the fMRI scan, participants played a virtual version of the ball-tossing game. Participants were told that they were playing the game with the other two participants; to maximize validity, photos of the other two participants were displayed during the game. Participants played three rounds of the ball-tossing game (initial inclusion, exclusion, re-inclusion). After the scan, participants filled out additional surveys, including measures assessing how distressed they felt during exclusion and manipulation check questions.

After the completion of the pre-scan questionnaire, participants were scanned using blood-oxygen-level-dependent (BOLD) fMRI while they completed two tasks, a resting-state scan, and structural MRI scans. In the task of interest to the current investigation, participants played a version of Cyberball, a virtual ball-tossing game that has been validated to elicit feelings of social exclusion in participants (Williams et al. 2000, Eisenberger et al. 2003, Hawkley et al. 2011, Cascio et al. 2015, Wang et al. 2017). Prior to playing the virtual game in the scanner, participants were given a cover story to encourage the belief that they were playing the game with the other two individuals who attended the study session with them, and each group of three participants practiced for the game that they would play inside the scanner by throwing a physical ball to one another prior to the scan. In an effort to further maximize task believability, participants also saw photos of the two other individuals from their village who also attended the session with them during the actual virtual ball-tossing game in the scanner. The Cyberball task consisted of three 2-minute rounds. In the first round (‘initial inclusion’), the other players in the game threw the ball equally to the participant and each other. In the second round (‘exclusion’), however, the two other players began to exclude the participant, throwing the ball exclusively to one another after a few fair throws. Finally, in the third round (‘re-inclusion’), the inverse of the exclusion round occurred, with the two other players exclusively throwing the ball to the participant after a few throws to one another. See Fig. 1 for an illustration of the task. Data from this task has recently been used to establish links between feelings of closeness among members of a triad and functional connectivity of brain regions implicated in social pain and mentalizing during social exclusion (Kim et al. 2023).

#### Post-scan self-report data

After participants completed the fMRI portion of the study, they also filled out post-scan questionnaires that asked them about their subjective experience during the Cyberball task. Participants were asked to indicate how ignored they felt during each round of Cyberball (e.g. ‘I felt ignored by the other players in round 1’); response options ranged from 1 (not at all) to 5 (very). They also indicated how sad they felt during round 2 (the exclusion round); response options for this item (‘I felt sad in round 2’) ranged from 1 (not at all) to 5 (very); this was the sole behavioural measure of distress during social exclusion. Participants also responded yes or no to two manipulation check items (‘I received fewer balls during the middle of round 2’ and ‘I felt that I was playing a computer game with the other participants, just like when playing a face-to-face ball-tossing game’); participants who indicated disagreement with either of these statements were excluded from analysis. In addition, we confirmed that no participants had knowledge of the deception prior to their participation. After completing the study session, participants were asked to refrain from discussing details of the study session with others in their community.

### fMRI image acquisition

Neuroimaging data were acquired using a 3 T Siemens Magnetom TrioTim scanner. Functional images were recorded using an interleaved slice sequence (180 volumes, TR = 2000 ms, TE = 30 ms, FOV = 240 × 240 mm, slice thickness = 4 mm, voxel size = 3.0 × 3.0 × 4.0 mm). High-resolution T1-weighted (T1w) images were acquired for each participant to be used for co-registration and normalization (224 slices, TR = 2300 ms, TE = 2.36 ms, FOV = 256 × 256 mm, slice thickness = 1 mm, voxel size = 1.0 × 1.0 × 1.0 mm).

### Imaging data analysis

Functional data were pre-processed using fMRIPrep 1.4.0 (Esteban et al. 2019), which is based on Nipype 1.2.0. The T1w image was corrected for intensity non-uniformity with N4BiasFieldCorrection, distributed with antsRegistration (ANTs 2.1.0; Avants et al. 2011), and used as T1w-reference throughout the workflow. Brain tissue segmentation of cerebrospinal fluid, white matter, and gray matter was performed on the brain-extracted T1w image using FSL (Functional Magnetic Resonance Imaging of the Brain (FMRIB) Software Library) fast (Smith et al. 2004). Volume-based spatial normalization to standard space (MNI152NLin2009cAsym) was performed through nonlinear registration with ANTs 2.1.0 (Avants et al. 2011), using brain-extracted versions of both the T1w reference and the T1w template.

For the BOLD run, the following preprocessing was performed. First, a reference volume and its skull-stripped version were generated using a custom methodology of fMRIPrep. The BOLD reference was then co-registered to the T1w reference using FSL flirt (Smith et al. 2004) with the boundary-based registration cost-function. Co-registration was configured with nine degrees of freedom to account for distortions remaining in the BOLD reference. Head-motion parameters with respect to the BOLD reference (transformation matrices and six corresponding rotation and translation parameters) were estimated before any spatiotemporal filtering using FSL mcflirt (Smith et al. 2004). The BOLD time-series were resampled onto their original, native space by applying a single, composite transform to correct for head-motion and susceptibility distortions. Then, the BOLD time-series were resampled into standard space (MNI152NLin2009cAsym).

### Task analysis

Data were modelled using the general linear model as implemented in nltools (Chang et al. 2019) in Python 3. We modelled three conditions, each corresponding to one round of Cyberball: initial inclusion, exclusion, and re-inclusion. A high-pass filter (128 s) was used to remove low-frequency noise. In addition, we included 24 nuisance regressors of no interest (six realignment parameters and the quadratic, derivative, and square of the derivate for each realignment parameter) to control for head motion. We also controlled for spikes in the data by identifying signals that exceeded the global mean signal by 3 SD and adding these as additional covariates of no interest to the design matrix. Finally, data were smoothed using a Gaussian kernel (6 mm full width at half maximum). Following past research (Eisenberger et al. 2003, Cacioppo et al. 2013, Cascio et al. 2015), we created a contrast to compare brain activity while participants experienced social exclusion compared to the first inclusion round, when they received the ball equally from other participants (exclusion > inclusion_round-1_). Percentage-signal-change scores were extracted for this contrast for each participant.

### Region-of-interest analysis

**Figure 2.**
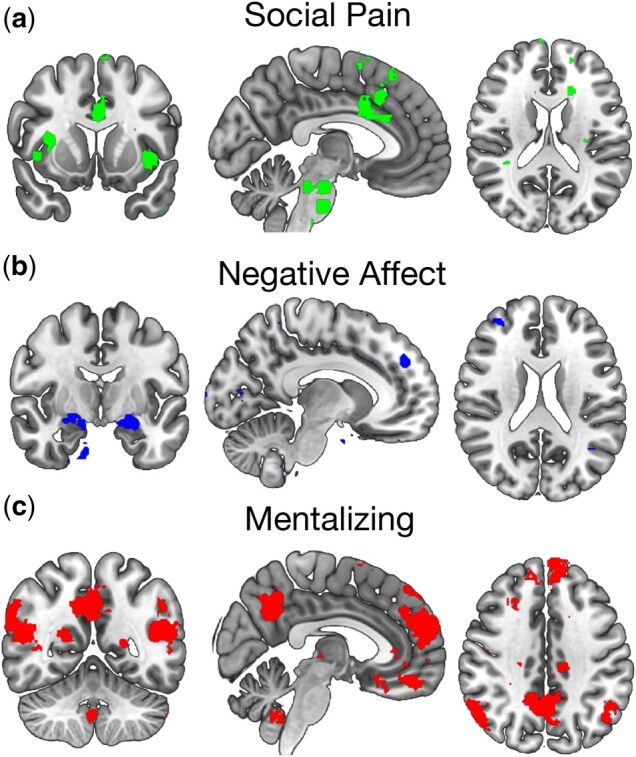
Regions of interest. Brain regions associated with (a) ‘social pain,’ (b) ‘negative affect,’ and (c) ‘mentalizing,’ as identified through Neurosynth using an association test (with a corrected *p*-value of <.01).

To investigate neural responses while participants experienced social exclusion, we conducted a series of analyses using neural activity extracted from three sets of *a priori* Region-of-interest (ROIs). We used Neurosynth (Yarkoni et al. 2011) to define targeted ROIs. Specifically, we used the ‘association test’ function to retrieve meta-analytic maps of the functional neuroimaging literature on ‘Social Pain,’ which consisted of subregions in the dorsal anterior cingulate (dACC) and bilateral anterior insula, ‘Negative Affect,’ which consisted of subregions in the bilateral amygdala and ACC, and ‘Mentalizing,’ which consisted of subregions in the medial prefrontal cortex, temporoparietal junction, precuneus, and right superior temporal sulcus (see Fig. 2). Neural activity in each of these three ROIs (social pain, negative affect, and mentalizing) was extracted for each participant for the contrast of interest (exclusion > inclusion_round-1_), resulting in one value per participant for each ROI representing the mean magnitude of activity across the voxels in the respective ROI during exclusion compared to inclusion. Outliers for the neural data were defined as values more than 1.5 interquartile ranges (IQRs) above the third quartile or below the first quartile. For each ROI, high mean activation values were recoded to a value equal to the upper quartile (i.e. 75th percentile) plus 1.5 times the IQR, and low mean activation values were recoded to a value equal to the lower quartile (i.e. 25th percentile) minus 1.5 times the IQR. All results reported in the main text are based on the data with recoded outliers (the pattern of results without recoded outliers is similar and is reported in the Supplemental Materials), and all analyses referring to brain activity in the ROIs reflect brain activity during exclusion compared to inclusion.

### Behavioural data analysis

**Figure 3.**
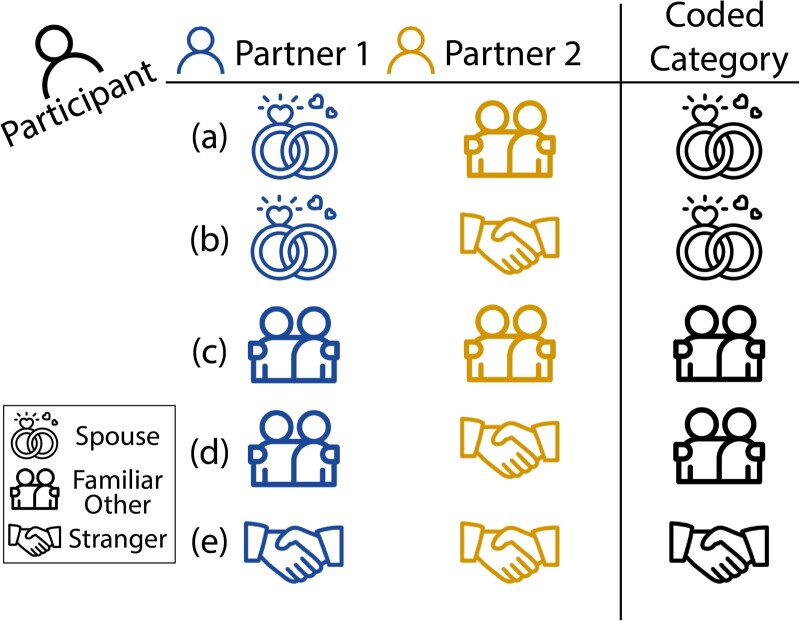
Recoding of the relationship variable. Each participant played Cyberball with two other participants (i.e. ‘partner 1’ and ‘partner 2’). Every possible combination of fMRI participants’ relationships with their two Cyberball partners are illustrated here (a–e). The relationship variable was defined by the more intimate relationship between the fMRI participant and their two partners. For instance, when an fMRI participant played with a spouse and a familiar other (a), they were coded as having played with a spouse.

To calculate a single measure of participants’ self-reported subjective emotional distress, we took the mean of participants’ self-reported feelings of being ignored and sad during social exclusion (i.e. round 2 of Cyberball). Furthermore, the two categorical variables measuring participants’ relationships with the other two Cyberball players (i.e. the other two participants who attended the same neuroimaging appointment and with whom participants believed they were playing Cyberball) were recoded for analysis using the following method. First, each variable was recoded for analysis such that friend, neighbour, and relative were grouped into a single ‘familiar other’ category, resulting in an ordered categorical variable with three factor levels: stranger, familiar other, and spouse. Then, for each participant, we created a new relationship variable by taking the more intimate relationship category from participants’ relationships with the two Cyberball players (with familiar other considered more ‘intimate’ than stranger, and spouse more ‘intimate’ than familiar other) to test whether the nature of participants’ relationship with the other players was associated with how distressed participants felt during exclusion (‘subjective emotional distress’), and whether this was related to their real-life social network centrality (Fig. 3). For example, if participant A attended the neuroimaging appointment with participant B (a spouse) and participant C (a friend), then participant A was coded to have played Cyberball with her spouse. Given that participant A and C are friends, if participant C was a stranger to participant B, then participant C was coded to have played Cyberball with a familiar other (i.e. as defined by her relationship with participant A). We determined that defining this relationship variable by the more intimate relationship category (of the relationships in the triad that involved the fMRI participant) was the most straightforward and meaningful way to capture individual differences in participants’ relationships with the other Cyberball players, as the more intimate relationship is likely to be more influential in participants’ qualitative experience in playing Cyberball given that a rejection from a close other is likely to be unexpected and particularly salient. Through categorizing the relationship variable by using the most intimate relationship type involving the fMRI participant, we can meaningfully differentiate whether the social rejection involves a close other or not (e.g. playing with two of one’s friends or with one friend and one stranger both involve rejection from a friend, and therefore is coded as playing with a familiar other; playing with two strangers, without the rejection from a familiar other, is coded as playing with a stranger). Furthermore, an approach of defining the relationship variable by every possible combination of participants’ relationships with the other two Cyberball players (i.e. five total possible combinations) would result in a much smaller number of observations per group, resulting in reduced power and interpretability. Thus, we opted to define this relationship variable based on the more intimate relationship of the two partners that participants played with as one meaningful way to test the effects of exclusion based on relationship type.

### Assessing links between neural correlates of emotional distress during social exclusion and social network centrality

We first investigated relationships between brain activity in our ROIs during exclusion, participants’ self-reported subjective emotional distress, and social network centrality. First, we tested whether the extent to which participants felt emotional distress during exclusion was associated with differences in brain activity in our three sets of ROIs (social pain, negative affective, and mentalizing) between social exclusion and inclusion. To do so, we fit linear regression models predicting self-reported subjective emotional distress from activity in each of our ROIs. Next, to determine whether the relationships between brain activity in our ROIs and subjective emotional distress were moderated by participants’ social network centrality, we tested the interaction between brain activity in each of our ROIs and out-degree centrality to predict subjective emotional distress. Given that participants could only nominate up to six people in the social network survey, it is possible that some participants have more than six people with whom they often discuss important matters with over the past year but were not given the space to nominate them. Therefore, we created an ordered categorical variable for analysis in hopes of capturing the full range of the out-degree centrality, such that the data was split into three approximately equally-sized ordered categories; specifically, an out-degree of six (*n *= 30) was classified as ‘high,’ an out-degree of five (*n *= 19) was classified as ‘medium,’ and an out-degree of four or lower were classified as ‘low’ (*n *= 24). We focus on comparing individuals in the ‘low’ and ‘high’ out-degree centrality categories in the current investigation, given that individuals belonging to these two categories are most likely to be qualitatively and meaningfully differentiated from one another than the individuals in the ‘medium’ out-degree centrality group. This categorical out-degree centrality variable that focused just on individuals in the ‘high’ and ‘low’ categories was used for all analyses using social network centrality that are reported in the main text; parallel analyses treating the out-degree centrality variable as continuous, including the ‘medium’ group, or splitting out-degree variable into two (rather than three) approximately equally-sized ordered categories (with an out degree of six classified as ‘high,’ *n *= 30, and an out-degree of five or lower classified as ‘low,’ *n *= 43) show similar results and are reported in the Supplemental Materials. While our main focus was on subjects’ perceived social connectedness as measured by their out-degree centrality, for completeness, we also ran analogous analyses using in-degree centrality and eigenvector centrality to explore their potential roles in social exclusion and report these results in the Supplemental Materials. All variables were z-scored prior to analysis, resulting in standardized regression coefficients.

### Assessing links between social network centrality and emotional distress during social exclusion

We next tested whether participants’ social network centrality was related to how distressed they reported feeling during exclusion. We conducted a pooled *t*-test to determine whether self-reported feelings of emotional distress during exclusion differed for participants with high versus low out-degree centrality. Next, we tested whether the relationship between social network centrality and subjective emotional distress was moderated by the status of participants’ relationships with the other players. To do so, we partitioned the data based on participants’ relationships with the other players (defined by the stronger relationship; see Fig. 3), which resulted in three non-overlapping subsets of data: 1) individuals whose closest partner in Cyberball was their spouse (*n *= 30), 2) individuals whose closest partner in Cyberball was a familiar other (*n *= 12), and 3) individuals who played Cyberball with two strangers (*n *= 12). We conducted an independent samples *t*-test in each of these subsets of data to test the relationship between out-degree centrality and subjective emotional distress within different levels of relationships with the other players. Analogous analyses were also conducted using in-degree centrality and eigenvector centrality, the results of which are included in the Supplemental Materials.

### Assessing the relationship between social network centrality and loneliness

In addition to our main analyses, given that prior work consistently suggests a negative association between social network centrality and subjective loneliness (Mullins and Dugan 1990, Hawkley et al. 2005, Shiovitz-Ezra and Leitsch 2010), we also tested the relationship between participants’ loneliness ratings and their out-degree centrality. We conducted this analysis on the larger sample of all subjects (*N *= 948) who completed the social network and loneliness questionnaires. We conducted a Kendall’s rank correlation to test the relationship between participants’ loneliness ratings and their out-degree centrality. We replicated this analysis on the neuroimaging subset of our sample. We report these results in Supplemental Information (Supplementary Fig. S1).

## Results

In the current investigation, we explored the relationships between participants’ real-life social network centrality and their neural and behavioural sensitivities to being socially excluded. We also tested whether these relationships would be affected by the nature of participants’ relationships with the other players, who had ostensibly excluded them during the ball-tossing game.

### Neural correlates of subjective emotional distress

**Figure 4.**
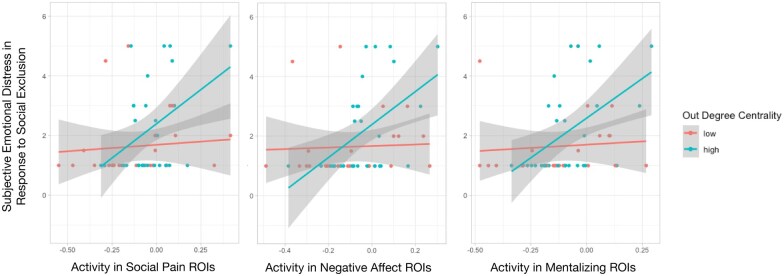
Associations between subjective distress and neural responses to social exclusion are driven by participants high in out-degree centrality. The positive associations between subjective emotional distress in response to social exclusion and activity in the social pain, negative affect, and mentalizing ROIs are driven by individuals with high out-degree centrality (i.e. who have many friends). Grey bands represent standard errors.

We first tested whether there was greater activity in our ROIs when participants self-reported greater subjective emotional distress at being excluded. Replicating prior work (Eisenberger et al. 2003, 2007, Dewall et al. 2012, Rotge et al. 2015), we found that mean activity in all three sets of ROIs was positively associated with subjective emotional distress. Greater activity in the social pain (*B *= 0.283, SE = 0.114, *p *= .016), negative affect (*B *= 0.307, SE = 0.113, *p *= .008), and mentalizing (*B *= 0.282, SE = 0.113, *p *= .015) ROIs was associated with greater subjective emotional distress during exclusion. We next tested whether this observed relationship was modulated by participants’ out-degree centrality. We found that the relationships between activity in all three sets of ROIs and subjective emotional distress were driven by participants with high out-degree centrality (Fig. 4), as indicated by significant interaction effects of out-degree centrality and ROI activity predicting subjective emotional distress (Tables 1–3). We note that in additional analyses treating out-degree centrality as continuous, the interaction effect between out-degree centrality and neural responding remained significant in the negative affect ROIs (*p *= .044; Table S5) and was marginally significant in the social pain ROIs (*p *= .069; Table S4), but was not significant in the mentalizing ROIs (*p *= .116; Table S6). Therefore, we urge caution in interpreting the significant interaction effects from our main analyses in the mentalizing ROIs (Table 3), and to a lesser extent, the social pain ROIs (Table 2).

**Table 1.** Predicting participants’ subjective emotional distress in response to social exclusion from mean activity in social pain ROIs, out-degree centrality, and their interaction.

Predictor	*B*	*SE*	*p*
Intercept	−0.194	0.193	.319
Activity in Social Pain ROIs	0.060	0.160	.709
Out-Degree Centrality	0.303	0.259	.247
Activity in Social Pain ROIs × Out-Degree Centrality	0.577	0.275	.041*

* *p* < .05

Note: Out-degree centrality is an ordered categorical variable with the reference level set to low; positive values indicate a greater association between high out-degree centrality and subjective emotional distress (the dependent variable).

**Table 2.** Predicting participants’ subjective emotional distress in response to social exclusion from mean activity in negative affect ROIs, out-degree centrality, and their interaction.

Predictor	*B*	*SE*	*p*
Intercept	−0.198	0.190	.300
Activity in Negative Affect ROIs	0.031	0.161	.848
Out-Degree Centrality	0.287	0.254	.264
Activity in Negative Affect ROIs × Out-Degree Centrality	0.671	0.262	.018*

* *p* < .05

Note: Out-degree centrality is an ordered categorical variable with the reference level set to low; positive values indicate a greater association between high out-degree centrality and subjective emotional distress (the dependent variable).

**Table 3.** Predicting participants’ subjective emotional distress in response to social exclusion from mean activity in mentalizing ROIs, out-degree centrality, and their interaction.

Predictor	*B*	*SE*	*p*
Intercept	−0.196	0.190	.305
Activity in Mentalizing ROIs	0.054	0.160	.737
Out-Degree Centrality	0.296	0.254	.249
Activity in Mentalizing ROIs × Out-Degree Centrality	0.604	0.262	.025*

* *p* < .05

Note: Out-degree centrality is an ordered categorical variable with the reference level set to low; positive values indicate a greater association between high out-degree centrality and subjective emotional distress (the dependent variable).

### Behavioural results

**Figure 5.**
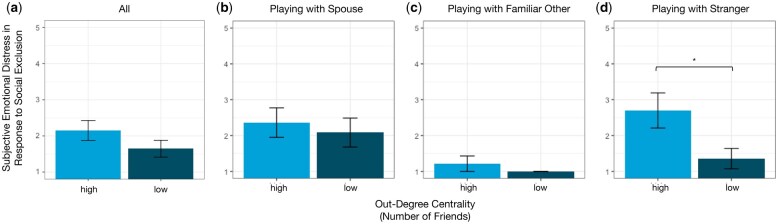
Subjective emotional distress in response to social exclusion by out-degree centrality. (a) There was a directional trend suggesting that participants with high out-degree centrality were overall more likely to self-report greater subjective distress during exclusion, compared to participants with low out-degree centrality, although this difference was not statistically significant at *p*< .05. (b–c) Similar to the overall results, although there was a directional trend suggesting that participants with high out-degree centrality were more likely to self-report greater subjective emotional distress when being excluded by a spouse or a familiar other, these differences were not statistically significant at p < .05. (d) When being excluded by two strangers, participants with high out-degree centrality self-reported significantly greater subjective emotional distress compared to participants with low out-degree centrality. Error bars represent standard errors; *p < .05.

We next conducted a pooled *t*-test to determine whether there was an association between individuals’ real-life social network centrality and the extent to which they felt distressed during social exclusion. We found that there was no statistically significant difference between people of high out-degree centrality (*M *= 2.150, SD = 1.521) and low out-degree centrality (*M *= 1.646, SD = 1.137) when self-reporting subjective distress during exclusion (*t*(52) = 1.349, *p *= .183) (Fig. 5a).

We next examined the relationship between subjective distress during social exclusion and out-degree centrality within subsets of data defined by participants’ relationships with their Cyberball partners. In participants who played Cyberball with their spouse and another person, no significant differences were found in the extent to which high out-degree centrality and low out-degree centrality participants self-reported feeling subjective distress during exclusion (*M*_high_ = 2.361, SD_high_ = 1.747; *M*_low_ = 2.083, SD_low_ = 1.395; *t*(28) = 0.461, *p *= .649). Similarly, no significant differences were found in the extent to which high out-degree centrality and low out-degree centrality participants reported feeling distress during exclusions when their closest Cyberball partner was a familiar other (*M*_high_ = 1.214, SD_high_ = 0.567; *M*_low_ = 1.000, SD_low_ = 0; *t*(6) = 1, *p *= .356; Welch’s *t*-test was performed given the samples had unequal variances). In participants who played with two strangers, however, we found that high out-degree participants self-reported greater distress during exclusion (*M *= 2.700, SD = 1.095) compared to low out-degree participants (*M *= 1.357, SD = 0.748), and this difference was statistically significant using a two-tailed *t-*test (*t*(10) = 2.539, *p *= .029). See Fig. 5b–d. We note that because these analyses focus on planned comparisons, they are not corrected for multiple comparisons; the results of additional analyses that were not planned *a priori* are reported in the Supplemental Materials. A similar pattern of results was observed when out-degree centrality was treated as a continuous variable and when we analysed the data using a linear regression model predicting self-reported distress during exclusion from out-degree centrality interacting with the most intimate relationship type followed by pairwise comparisons (see Supplementary Fig. S3, Table S13, and Table S14).

Given the finding that people with high out-degree centrality show behavioural sensitivity to exclusion when playing with strangers, but not when playing with their spouse or a familiar other, we conducted additional analyses exploring the relationship between neural sensitivity to exclusion and out-degree centrality when playing with different Cyberball partners (spouse, familiar other, or stranger). Indeed, we found that the association between neural sensitivity in the social pain and negative affect ROIs to social exclusion and out-degree centrality was most robust when participants were playing with strangers (see Supplementary Fig. S4).

Finally, as an exploratory analysis, we tested the relationship between participants’ loneliness ratings and their out-degree centrality. In line with past research (Mullins and Dugan 1990, Hawkley et al. 2005, Shiovitz-Ezra and Leitsch 2010), loneliness was negatively associated with out-degree centrality (see Supplementary Fig. S1).

## Discussion

We found support for the hypothesis that people who have high out-degree centrality (i.e. who have many friends) show greater sensitivity to social exclusion, even though they are less lonely overall. Neural responses in brain regions implicated in sensitivity to social exclusion—social pain, negative affect, and mentalizing sets of ROIs—scaled with subjective distress during exclusion, but only in highly-central individuals. Furthermore, highly-central individuals self-reported greater distress when excluded by strangers, compared to less-central individuals (i.e. individuals with fewer friends), and similar patterns were found in the brain data, such that in highly-central individuals, neural sensitivity to exclusion in the social pain and negative affect sets of ROIs was most pronounced when being excluded by strangers. Accordingly, findings suggest that while perceiving oneself to have many social connections is associated with less loneliness overall, it may be linked to greater distress in certain contexts, such as being excluded by strangers. Our work contributes to the understanding of how features of individuals’ real-life social worlds, such as perceived numbers of social ties, are associated with responses to negative social experiences, such as exclusion.

Our findings illustrate that highly-central individuals may be more sensitive to social exclusion. Replicating prior work (Eisenberger et al. 2003, Dewall et al. 2012, Rotge et al. 2015), in the current study, increased activity in all three sets of ROIs—social pain, negative affect, and mentalizing—was associated with greater distress during exclusion; however, this association was only observed in highly-central individuals. Thus, increased activity in the social pain, negative affect, and mentalizing sets of ROIs may be particularly reflective of distress in individuals who are highly central in their social network, but not in less-central individuals. In prior work, rejection sensitivity moderated activity in the dACC, a subregion of the social pain ROI, while participants viewed disapproving facial expressions (Burklund et al. 2007). Thus, one possibility is that in highly-central individuals, responses in such brain regions track socially threatening situations particularly closely. In contrast, in *less*-central individuals, neural responding in these functionally heterogeneous ROIs was not associated with distress during exclusion, and thus, may be supporting other mental processes (e.g. mind-wandering). Accordingly, there may be individual differences with regard to the degree to which we can decode psychological states from brain activity (i.e. reverse inference) (Poldrack 2011) during social exclusion.

Further, even though highly-central individuals are found to be less lonely overall, replicating findings from previous work (Russell et al. 1984, House et al. 1988), self-report and neural results from the current study suggest that highly-central individuals exhibit greater sensitivity to exclusion by strangers. Specifically, we found that highly-central individuals were most sensitive to rejection by new social partners, suggesting that the association between social network centrality and rejection sensitivity is context-dependent (e.g. highly-central individuals may be less sensitive to rejection by people with whom they have established social ties, but more sensitive to rejection by new potential social partners). This observation extends previous findings by highlighting that features (e.g. having many social ties) generally thought to promote well-being may actually be associated with a higher risk for distress related to disconnection in certain contexts. For example, although quantity of social ties has been associated with reduction in rejection sensitivity in children and adolescents in general (Bagwell et al. 2001, Masten et al. 2012), our results suggest that contexts may shape rejection sensitivity in specific ways (e.g. being rejected by a stranger in a densely connected village may be more salient). That said, we acknowledge that there may be methodological differences in experimental paradigms (e.g. how centrality was operationalized, how rejection sensitivity was assessed, the context of rejection, etc) and broader differences in how people belonging to different age groups perceive and respond to rejection. In addition, this heightened rejection sensitivity in highly-central individuals could have negative consequences, since greater behavioural and neural rejection sensitivity has been linked to antisocial behaviours, such as aggression and anxious attachment (Dewall et al. 2012, Beyer et al. 2014).

Why do people with more friends exhibit heightened sensitivity to rejection by strangers? One possibility is that highly-central individuals have a greater need to be accepted by many people and therefore are more sensitive to potential threats to meeting this need (Pickett et al. 2004). In other words, the same traits (e.g. high need-to-belong) that drive the highly-central people to forge and maintain many social ties may also drive them to be more sensitive to rejection by new potential partners, and likewise, the same traits that drive less-central people to have fewer social ties may drive them to be indifferent to being rejected by strangers. Therefore, highly-central individuals may show selectively greater sensitivity to rejection by strangers, given that strangers are potential new connections that can help meet their higher need to belong. This may be particularly true in the current study, since all participants were from the same village where future interactions between strangers may be exceptionally likely. It is also possible that this heightened sensitivity to social rejection reflects an adaptive tendency that can promote long-term well-being, even if it engenders short-term distress in certain contexts. One theory suggests that similar to physical pain, social pain is a critical adaptive mechanism that alerts us of potential threats to social connection and protects us from further social disconnection (Eisenberger and Lieberman 2004, MacDonald and Leary 2005). In line with the theory, being able to recognize and respond quickly to social threats may be a pre-requisite for people to assume a central position within their social networks by increasing their proclivity to establish or restore connection, leading to less loneliness overall and thus promoting long-term well-being. Additionally, highly-central individuals may be especially motivated to distinguish between the people with whom they are playing and attribute the rejection behaviours to different causes (e.g. they may attribute rejection when playing with spouses or other familiar individuals to external factors, such as their spouse wanting to make the other player feel included, and attribute rejection when playing with strangers to personal reasons, such as their own behaviors and how others think of them), and thus become more sensitive to rejection by strangers. Another possibility is that activity in our ROIs—specifically, in the dACC—during exclusion reflects an expectancy violation in highly-central individuals (Somerville et al. 2006); individuals with many friends may have greater expectations of being socially included by strangers, perhaps because they are accustomed to being widely accepted by others in everyday life. Future work can arbitrate between these possibilities and further develop theories linking real-world social features and rejection sensitivity.

In the current study, we characterized the triad for each fMRI participant based on the closest relationship that they had with other members of the triad, reasoning that this would be most relevant to participants’ experience of social rejection. That said, we note that it is also possible that the existence of a specific type of relationship within the triad (whether or not that relationship involves the participant in question) may influence aspects of participants’ experience in the study. For example, a triad that included a spouse or familiar-other pair may experience more positive affect when they were becoming familiar with the Cyberball paradigm (i.e. when they were throwing a physical ball to one another outside the scanner before being scanned), which may subsequently influence people’s experience during social exclusion. Future research could explore this possibility by assessing and controlling for affective states prior to scanning. Furthermore, since each triad is composed of three dyadic relationships, it is also possible that different types of relationships within a triad (e.g. a spouse pair with a mutual friend, a spouse pair with a friend to only one of the spouses, and a spouse pair with a stranger) may interact to shape how people respond to social rejection. We hope that future work with a larger sample size may be able to assess these more fine-grained interactions among different types of relationships and their influence on social rejection.

In addition, while we find that perceiving oneself to have more friends is associated with feeling less lonely overall, it may be interesting to explore how loneliness may interact with one’s real-world social network to influence rejection sensitivity. For example, future research can investigate whether people who perceive themselves to have many connections but feel lonely are more sensitive to social rejection because they feel insecure about existing relationships. Further, the current study focused on investigating the relationship between people’s responses to social rejection and out-degree centrality, which captures one’s perception of social connectedness and may be informed by one’s subjective experiences with others and relate to one’s ability and proclivity to access resources, such as social support, in the network. Our exploratory analyses on in-degree centrality and eigenvector centrality, which rely on sociocentric network data and may be indicative of an individual’s popularity and status, yielded null results, suggesting that rejection sensitivity may be largely influenced by an individual’s perception of their own social ties. However, future work should continue to explore potential mechanisms that may mediate or moderate the relationship between perceived social connectedness and rejection sensitivity.

Notably, our sample consisted of older adults living in a South Korean village. Given that much of the existing literature in Psychology has disproportionally focused on WEIRD (White, Educated, Industrialized, Rich, and Democratic) samples that severely limit the generalizability of findings (Arnett 2008, Dan 2010), our findings based on a non-WEIRD sample can deepen understanding of the interplay between social network characteristics and basic psychological and neural processes involved in social rejection. Our findings shed light on these processes in a high-risk population for social disconnection; loneliness and isolation are highly prevalent in older adult populations across the world, with devastating consequences (Shankar et al. 2011, Walsh et al. 2017). We acknowledge that there are several methodological limitations in our study, including limiting the self-report of social ties to a maximum of 6, as well as having rather small sample sizes per condition, low statistical power, and a focus on planned, ROI-focused analyses without correction for multiple comparisons. Nonetheless, we believe that these findings from this unique dataset consisting of a sociocentric network of older adults within a bounded community in South Korea are informative to motivate future research on social exclusion, especially taking the context of exclusion into account, in non-WEIRD samples and other populations at high risk for social disconnection. In particular, future work using a similar paradigm to that used in the current study can elucidate the generalizability of our findings with larger numbers of participants per condition and in different developmental samples and cultures, since there may be cultural differences in brain activity during social processes (Han and Ma 2014) and age-related differences in exclusion reactivity (Löckenhoff et al. 2013, Vijayakumar et al. 2017), as well as differences across cultures and stages of development in more general social phenomena (e.g. relational mobility). Studying such phenomena over the course of development is particularly important, given the increasing prevalence of anonymous online interactions between strangers (e.g. anonymous cyberbullying).

In summary, these findings suggest that people who have many friends show greater neural sensitivity to social exclusion, and greater behavioral sensitivity to being excluded by potential new social ties, despite reporting less overall subjective social disconnection (i.e. loneliness). Future work can elucidate the mechanisms underlying these effects and whether this social sensitization is adaptive or maladaptive over time. Taken together, our findings provide a nuanced picture of centrality in social networks, which has largely been linked to beneficial outcomes for the individual, and offer insight into how real-world social network features are associated with neural and behavioral reactivity to social experiences.

## Supplementary Material

nsaf067_Supplementary_Data

## Data Availability

The data will be shared upon reasonable request to the corresponding authors.

## Statement of relevance

Humans have a fundamental need to socially belong. Indeed, prior work suggests that individuals show psychological and neural sensitivity to being excluded by social partners. However, little is known about how features of someone’s real-life social world affect the way that they respond during social interactions. We tested for links between the number of friends that a person has and their sensitivity to social exclusion. Although well-connected people reported *less* overall loneliness, neural and behavioral evidence suggested that they experienced *greater* distress when being socially excluded by strangers. These findings suggest that factors often thought of as protective against feelings of social disconnection could actually confer greater distress in certain situations. We discuss the possible adaptive value of this social sensitization among well-connected people. More broadly, these findings demonstrate the utility of combining characterizations of people’s real-life social worlds and their mental processing to advance understanding of human sociality.
