# Supplementary material for: Having more friends is associated with greater sensitization to social exclusion: neural and behavioral evidence
Source: Soc Cogn Affect Neurosci. 2025 Jun 30;20(1):nsaf067. doi: 10.1093/scan/nsaf067 (PMC12342143; doi:10.1093/scan/nsaf067)
Supplement: nsaf067_Supplementary_Data [file nsaf067_supplementary_data.docx]

Supplemental Information

**Social Network Centrality and Loneliness**

We tested whether there was an association between individuals’ social network centrality and overall feelings of loneliness in everyday life. In line with past research (Hawkley et al., 2005; Mullins & Dugan, 1990; Shiovitz-Ezra & Leitsch, 2010), loneliness was negatively associated with out-degree centrality in the full sample, both when treating out-degree centrality as a continuous variable (tau = -0.110, z = -3.862, *p* < 0.001) and when contrasting the high (*M*_loneliness_ = 0.220, SD_loneliness_ = 0.564) and low (*M*_loneliness_ = 0.414, SD_loneliness_ = 0.766) centrality groups (*t*(736.7) = 3.965, *p* < 0.001; Fig. S1a)^[[1]](#footnote-1)^. The same pattern of results was found in the subset of participants who were scanned, both when treating out-degree centrality as a continuous variable (tau = -0.223, z = -2.094, *p* = 0.036) and when contrasting the high (*M*_loneliness_ = 0.032, SD_loneliness_ = 0.180) and low (*M*_loneliness_ = 0.333, SD_loneliness_ = 0.702) centrality groups (*t*(25.34) = 2.050, *p* = 0.051; Fig. S1b).


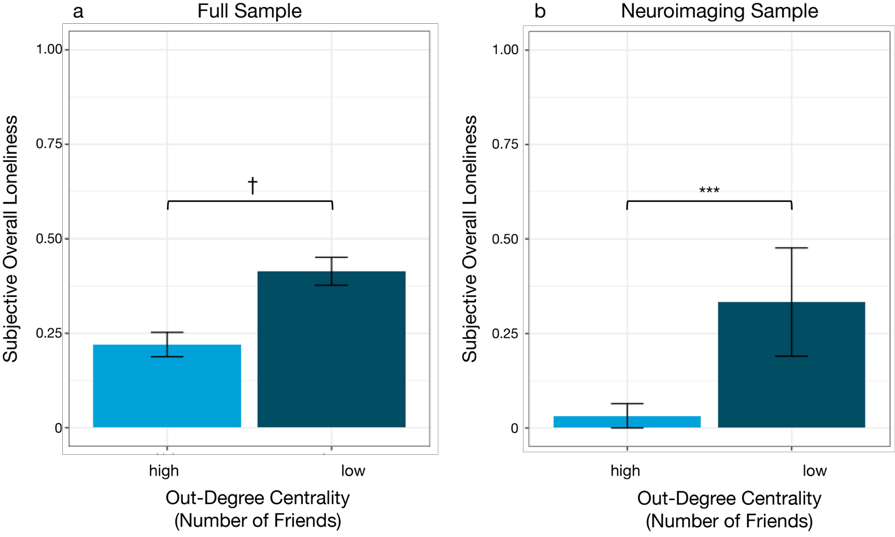


**Fig. S1. Out-degree centrality and subjective overall loneliness.** Participants with high out-degree centrality were less likely to report experiencing overall loneliness compared to participants with low out-degree centrality in both the (a) full sample and (b) neuroimaging sample. Similar results were found when treating out-degree centrality as continuous variable in both the full (*p* < 0.001) and neuroimaging (*p* = 0.036) samples. Error bars represent standard errors. ****p* < 0.001, †*p* < 0.10.


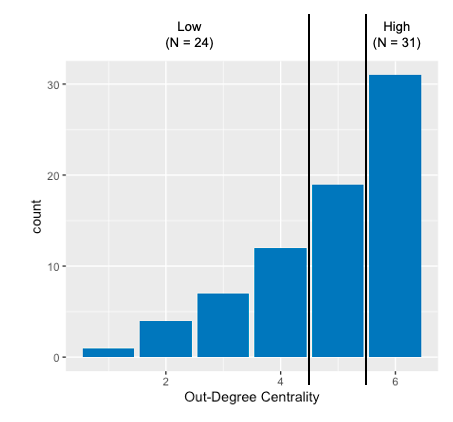


**Fig. S2. Distribution of out-degree centrality*.*** The vertical black lines indicate the thresholds used to divide the full sample into 3 approximately equally-sized groups.

**Neural Correlates of Subjective Emotional Distress – Including Outliers**

Here, we fit additional linear regression models that are analogous to the results reported in the “Neural Correlates of Subjective Emotional Distress” section in the main manuscript, except that we did not recode the outliers. Similar to what we reported in the main manuscript, mean activity in all three sets of ROIs was positively associated with subjective emotional distress. Greater activity in the social pain (*B* = 0.280, SE = 0.114, *p* = 0.017), negative affect (*B* = 0.287, SE = 0.113, *p* = 0.014), and mentalizing (*B* = 0.282, SE = 0.113, *p* = 0.015) ROIs was associated with greater subjective emotional distress during exclusion.

We also tested whether this observed relationship was modulated by participants’ out-degree centrality, using the non-recoded dataset. Similar to the results that we reported in the main manuscript, we found that the relationships between activity in all 3 sets of ROIs and subjective emotional distress were driven by participants with high out-degree centrality, as indicated by significant interaction effects of out-degree centrality and ROI activity predicting subjective emotional distress (Tables S1-S3).

Table S1. Predicting participants’ subjective emotional distress in response to social exclusion from mean activity in social pain ROIs, out-degree centrality, and their interaction

| Predictor | *B* | *SE* | *p* |  |
| --- | --- | --- | --- | --- |
| Intercept | -0.189 | 0.193 | 0.331 |  |
| Activity in Social Pain ROIs | 0.075 | 0.150 | 0.619 |  |
| Out-Degree Centrality | 0.261 | 0.259 | 0.318 |  |
| Activity in Social Pain ROIs*Out-Degree Centrality | 0.673 | 0.297 | 0.028* |  |
| **p* <.05, ***p* <.01, ****p* <.001 | | | | |

Note: out-degree centrality is a categorical variable with the reference level set to low; positive values indicate greater association between high out-degree centrality and subjective emotional distress (the dependent variable).

Table S2. Predicting participants’ subjective emotional distress in response to social exclusion from mean activity in negative affect ROIs, out-degree centrality, and their interaction

| Predictor | *B* | *SE* | *p* |  |
| --- | --- | --- | --- | --- |
| Intercept | -0.193 | 0.190 | 0.315 |  |
| Activity in Negative Affect ROIs | 0.054 | 0.147 | 0.715 |  |
| Out-Degree Centrality | 0.240 | 0.256 | 0.352 |  |
| Activity in Negative Affect ROIs*Out-Degree Centrality | 0.777 | 0.294 | 0.011* |  |
| **p* <.05, ***p* <.01, ****p* <.001 | | | | |

Note: out-degree centrality is a categorical variable with the reference level set to low; positive values indicate greater association between high out-degree centrality and subjective emotional distress (the dependent variable).

Table S3. Predicting participants’ subjective emotional distress in response to social exclusion from mean activity in mentalizing ROIs, out-degree centrality, and their interaction

| Predictor | *B* | *SE* | *p* |  |
| --- | --- | --- | --- | --- |
| Intercept | -0.191 | 0.189 | 0.318 |  |
| Activity in Mentalizing ROIs | 0.070 | 0.150 | 0.643 |  |
| Out-Degree Centrality | 0.251 | 0.255 | 0.329 |  |
| Activity in Mentalizing ROIs*Out-Degree Centrality | 0.700 | 0.280 | 0.016* |  |
| **p* <.05, ***p* <.01, ****p* <.001 | | | | |

Note: out-degree centrality is a categorical variable with the reference level set to low; positive values indicate greater association between high out-degree centrality and subjective emotional distress (the dependent variable).

**Neural Correlates of Subjective Emotional Distress Using Uncategorized (i.e., Continuous) Out-Degree Centrality**

We also fit additional linear regression models to predict subjective emotional distress from the interaction of out-degree centrality and brain activity in the three ROIs using the continuous out-degree centrality variable. Results indicate similar patterns as those reported in the main manuscript; associations between neural responses and subjective emotional distress tended to be stronger for individuals with high out-degree centrality (see Tables S4-S6). However, as noted in the main text, although the interaction between out-degree centrality and neural responding remained significant in the negative affect ROIs (see Table S5) and marginally significant in the social pain ROIs (see Table S4) in these analyses, the interaction was not significant in the mentalizing ROIs (see Table S6). Therefore, some of the interaction effects from our primary analyses reported in the main text – in particular, that involving the mentalizing ROIs, and to a lesser extent, that involving the social pain ROIs – should be interpreted with caution.

Table S4. Predicting participants’ subjective emotional distress from mean activity in social pain ROI, out-degree centrality, and their interaction

| Predictor | *B* | *SE* | *p* |  |
| --- | --- | --- | --- | --- |
| Intercept | -0.031 | 0.113 | 0.786 |  |
| Activity in Social Pain ROI | 0.319 | 0.119 | 0.009** |  |
| Out-Degree Centrality | 0.201 | 0.116 | 0.089† |  |
| Activity in Social Pain ROI*Out-Degree Centrality | 0.203 | 0.110 | 0.069† |  |
| †*p* < 0.10, **p* <.05, ***p* <.01, ****p* <.001 | | | | |

Table S5. Predicting participants’ subjective emotional distress from mean activity in negative affect ROI, out-degree centrality, and their interaction

| Predictor | *B* | *SE* | *p* |  |
| --- | --- | --- | --- | --- |
| Intercept | -0.041 | 0.112 | 0.716 |  |
| Activity in Negative Affect ROI | 0.336 | 0.116 | 0.005** |  |
| Out-Degree Centrality | 0.201 | 0.116 | 0.089† |  |
| Activity in Negative Affect ROI*Out-Degree Centrality | 0.214 | 0.105 | 0.044* |  |
| **p* <.05, ***p* <.01, ****p* <.001 | | | | |

Table S6. Predicting participants’ subjective emotional distress from mean activity in mentalizing ROI, out-degree centrality, and their interaction

| Predictor | *B* | *SE* | *p* |  |
| --- | --- | --- | --- | --- |
| Intercept | -0.029 | 0.114 | 0.800 |  |
| Activity in Mentalizing ROI | 0.301 | 0.117 | 0.013* |  |
| Out-Degree Centrality | 0.195 | 0.117 | 0.102 |  |
| Activity in Mentalizing ROI*Out-Degree Centrality | 0.168 | 0.106 | 0.116 |  |
| **p* <.05, ***p* <.01, ****p* <.001 | | | | |

**Neural Correlates of Subjective Emotional Distress – Including the “Medium” Group**

We also fit additional linear regression models to predict subjective emotional distress from the interaction of out-degree centrality and brain activity in the three ROIs using the out-degree centrality variable when the “medium” group (i.e., having 5 ties) was included. Results indicate similar patterns as those reported in the main manuscript, with the associations between activity in the brain ROIs and subjective emotional distress being stronger for individuals with high out-degree centrality when compared to individuals with low out-degree centrality (see Tables S7-S9).

Table S7. Predicting participants’ subjective emotional distress in response to social exclusion from mean activity in social pain ROIs, out-degree centrality, and their interaction

| Predictor | *B* | *SE* | *p* |
| --- | --- | --- | --- |
| Intercept | -0.225 | 0.195 | 0.252 |
| Activity in Social Pain ROIs | 0.062 | 0.159 | 0.699 |
| Out-Degree Centrality (high) | 0.304 | 0.262 | 0.251 |
| Out-Degree Centrality (medium) | 0.296 | 0.297 | 0.323 |
| Activity in Social Pain ROIs*Out-Degree Centrality (high) | 0.593 | 0.273 | 0.033* |
| Activity in Social Pain ROIs*Out-Degree Centrality (medium) | 0.229 | 0.284 | 0.423 |

**p* <.05, ***p* <.01, ****p* <.001

Note: Out-degree centrality is an ordered categorical variable with three levels: high (having 6 or more ties; N = 30) and medium (having 5 ties; N = 19) and low (having 4 or fewer ties; N = 24). The reference level for out-degree centrality is set to low. Positive values indicate a greater association between high out-degree centrality and subjective emotional distress (the dependent variable).

Table S8. Predicting participants’ subjective emotional distress in response to social exclusion from mean activity in negative affect ROIs, out-degree centrality, and their interaction

| Predictor | *B* | *SE* | *p* |
| --- | --- | --- | --- |
| Intercept | -0.229 | 0.192 | 0.238 |
| Activity in Negative Affect ROIs | 0.032 | 0.163 | 0.843 |
| Out-Degree Centrality (high) | 0.289 | 0.258 | 0.266 |
| Out-Degree Centrality (medium) | 0.294 | 0.292 | 0.317 |
| Activity in Negative Affect ROIs*Out-Degree Centrality (high) | 0.668 | 0.264 | 0.014* |
| Activity in Negative Affect ROIs*Out-Degree Centrality (medium) | 0.273 | 0.274 | 0.322 |

**p* <.05, ***p* <.01, ****p* <.001

Note: Out-degree centrality is an ordered categorical variable with three levels: high (having 6 or more ties; N = 30) and medium (having 5 ties; N = 19) and low (having 4 or fewer ties; N = 24). The reference level for out-degree centrality is set to low. Positive values indicate a greater association between high out-degree centrality and subjective emotional distress (the dependent variable).

Table S9. Predicting participants’ subjective emotional distress in response to social exclusion from mean activity in mentalizing ROIs, out-degree centrality, and their interaction

| Predictor | *B* | *SE* | *p* |
| --- | --- | --- | --- |
| Intercept | -0.228 | 0.193 | 0.242 |
| Activity in Mentalizing ROIs | 0.055 | 0.158 | 0.730 |
| Out-Degree Centrality (high) | 0.288 | 0.260 | 0.278 |
| Out-Degree Centrality (medium) | 0.310 | 0.296 | 0.298 |
| Activity in Mentalizing ROIs*Out-Degree Centrality (high) | 0.612 | 0.258 | 0.021* |
| Activity in Mentalizing ROIs*Out-Degree Centrality (medium) | 0.167 | 0.294 | 0.569 |

**p* <.05, ***p* <.01, ****p* <.001

Note: Out-degree centrality is an ordered categorical variable with three levels: high (having 6 or more ties; N = 30) and medium (having 5 ties; N = 19) and low (having 4 or fewer ties; N = 24). The reference level for out-degree centrality is set to low. Positive values indicate a greater association between high out-degree centrality and subjective emotional distress (the dependent variable).

**Neural Correlates of Subjective Emotional Distress – Transforming out-degree centrality into two (rather than three) approximately equal-sized groups**

We also fit additional linear regression models to predict subjective emotional distress from the interaction of out-degree centrality and brain activity in the three ROIs using the out-degree centrality variable transformed into two, rather than 3, approximately equal-sized groups (high out-degree centrality: having 6 or more ties, *n* = 30; low out-degree centrality: having 1-5 ties, *n* = 43). Results indicate similar patterns as those reported in the main manuscript, with the associations between activity in the brain ROIs and subjective emotional distress being stronger for individuals with high out-degree centrality (see Tables S10-S12).

Table S10. Predicting participants’ subjective emotional distress in response to social exclusion from mean activity in social pain ROIs, out-degree centrality, and their interaction

| Predictor | *B* | *SE* | *p* |
| --- | --- | --- | --- |
| Intercept | -0.095 | 0.147 | 0.518 |
| Activity in Social Pain ROIs | 0.138 | 0.131 | 0.295 |
| Out-Degree Centrality | 0.174 | 0.228 | 0.449 |
| Activity in Social Pain ROIs*Out-Degree Centrality | 0.517 | 0.257 | 0.048* |

**p* <.05, ***p* <.01, ****p* <.001

Note: Out-degree centrality is an ordered categorical variable with two levels: high (having 6 or more ties; N = 30) and low (having 1-5 ties; N = 43). The reference level for out-degree centrality is set to low. Positive values indicate a greater association between high out-degree centrality and subjective emotional distress (the dependent variable).

Table S11. Predicting participants’ subjective emotional distress in response to social exclusion from mean activity in negative affect ROIs, out-degree centrality, and their interaction

| Predictor | *B* | *SE* | *p* |
| --- | --- | --- | --- |
| Intercept | -0.095 | 0.144 | 0.512 |
| Activity in Negative affect ROIs | 0.136 | 0.131 | 0.303 |
| Out-Degree Centrality | 0.156 | 0.225 | 0.491 |
| Activity in Negative Affect ROIs*Out-Degree Centrality | 0.565 | 0.245 | 0.024* |

**p* <.05, ***p* <.01, ****p* <.001

Note: Out-degree centrality is an ordered categorical variable with two levels: high (having 6 or more ties; N = 30) and low (having 1-5 ties; N = 43). The reference level for out-degree centrality is set to low. Positive values indicate a greater association between high out-degree centrality and subjective emotional distress (the dependent variable).

Table S12. Predicting participants’ subjective emotional distress in response to social exclusion from mean activity in mentalizing ROIs, out-degree centrality, and their interaction

| Predictor | *B* | *SE* | *p* |
| --- | --- | --- | --- |
| Intercept | -0.098 | 0.145 | 0.503 |
| Activity in Mentalizing ROIs | 0.103 | 0.132 | 0.441 |
| Out-Degree Centrality | 0.155 | 0.226 | 0.497 |
| Activity in Mentalizing ROIs*Out-Degree Centrality | 0.564 | 0.242 | 0.023* |

**p* <.05, ***p* <.01, ****p* <.001

Note: Out-degree centrality is an ordered categorical variable with two levels: high (having 6 or more ties; N = 30) and low (having 1-5 ties; N = 43). The reference level for out-degree centrality is set to low. Positive values indicate a greater association between high out-degree centrality and subjective emotional distress (the dependent variable).

**Behavioral Results Using Uncategorized (i.e., Continuous) Out-Degree Centrality**

We ran parallel analyses to those reported in the main manuscript to determine the relationship between the continuous out-degree centrality variable and subjective emotional distress. Given the highly skewed nature of the out-degree centrality variable, we used Kendall’s rank correlation to compute the relationship between the continuous out-degree centrality variable and subjective emotional distress. Similar to the main results reported in the manuscript, we found that participants with higher out-degree centrality were directionally more likely to report greater subjective emotional distress at being excluded, although this relationship was not statistically significant (tau = 0.116, z = 1.172, *p* = 0.241).

We next tested whether this relationship was affected by the nature of participants’ relationships with the other two players. To do so, we conducted 3 additional Kendall’s rank correlations on data that were subset based on participants’ relationships with the other players (i.e., analogous to what we did in the main analyses). Paralleling the main results, we found that the relationship between out-degree centrality and subjective emotional distress was only found in participants who played with strangers (see Figure S3). We also ran linear models to predict subjective emotional distress using participants’ relationships with other players, continuous out-degree centrality, and their interaction, which yielded similar results as mentioned above and in the main analyses: when playing with strangers, the relationship between subjective emotional distress and out-degree centrality is significant (*B =* 0.432, *SE =* 0.213, *p =* 0.043); the relationship between subjective emotional distress and out-degree centrality is not significant when playing with a pair that includes a friend (*B =* 0.0756, *SE =* 0.244, *p =* 0.757) or a spouse (*B =* 0.124, *SE =* 0.174, *p =* 0.478).


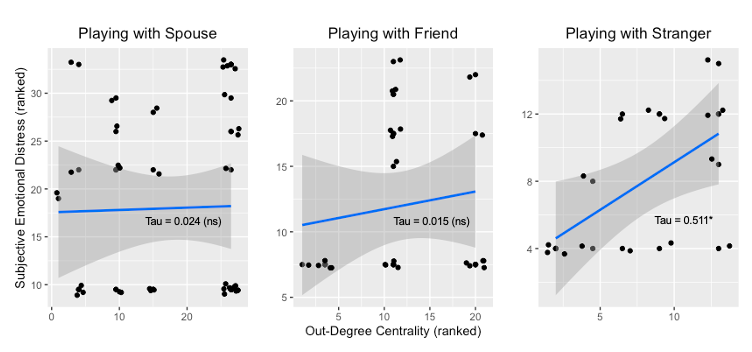


**Figure S3. Relationship between out-degree centrality and subjective emotional distress by participants’ relationships with other players***.* Paralleling the main results, there was a positive association between out-degree centrality and subjective emotional distress when participants played with two strangers, but not when they played with a spouse or a friend. Grey bands represent standard errors, **p* < 0.05.

**Behavioral Results Using Out-Degree Centrality Transformed into two (rather than three) approximately equal-sized groups**

We ran the same analyses as reported in the main manuscript, with out-degree centrality transformed into two, rather than three, approximately equal-sized groups (high out-degree centrality: having 6 or more ties, *n* = 30; low out-degree centrality: having 1-5 ties, *n* = 43). We conducted a pooled t-test comparing the high and low out-degree centrality groups to investigate whether there was an association between individuals’ real-life social network centrality and the extent to which they felt distressed during social exclusion. Replicating the pattern of results observed in the manuscript, we did not observe a statistically significant relationship between out-degree centrality and self-reported distress during exclusion overall (*t*(70) = 1.090, *p* = 0.280). Then, we conducted independent samples t-tests within each of the categories defined by the more intimate relationships the participants have in the triad. Similar to the patterns of results reported in the manuscript, while no significant differences were found in the extent to which high out-degree centrality and low out-degree centrality participants self-reported feeling subjective distress during exclusion when playing with their spouse and someone else (*t*(33) = 0.748, *p* = 0.460) or when at least one of the player is a familiar other (*t*(20) = -1.168, *p* = 0.257), a marginally significant difference was observed when participants played with both strangers (*t*(13) = 1.930, *p* = 0.076).

**Behavioral Results Using a Linear Regression Model Predicting Subjective Distress With Closest Relationship Type, Out-degree Centrality, and Their Interaction**

We ran a linear regression model and paired comparison analyses to probe the differences between people of high versus low out-degree centrality within each relationship type. Consistent with the results reported in the manuscript, no significant differences were found between participants of low versus high out-degree centrality in their self-reported level of distress when their closest Cyberball partner was a spouse (*M*_low_ - *M*_high_ = -0.202, SE = 0.357, *p* = 0.574) or familiar other (*M*_low_ - *M*_high_ = -0.156, SE = 0.561, *p* = 0.783). For participants who played with two strangers, there exists a marginally significant difference such that people of high out-degree centrality self-report higher levels of distress when experiencing social exclusion (*M*_low_ – *M*_high_ = -0.977, SE = 0.561, *p* = 0.0883).

**Table S13. Linear model predicting subjective distress using the most intimate relationship type, out-degree centrality, and their interaction**

| Predictor | *B* | *SE* | *p* |
| --- | --- | --- | --- |
| Intercept | -0.414 | 0.362 | 0.259 |
| Relationship type (familiar other) | -0.260 | 0.561 | 0.646 |
| Relationship type (spouse) | 0.528 | 0.456 | 0.252 |
| Out-Degree Centrality (high) | 0.977 | 0.561 | 0.088 |
| Relationship type (familiar other)*Out-Degree Centrality (high) | -0.821 | 0.794 | 0.306 |
| Relationship type (spouse)*Out-Degree Centrality (high) | -0.774 | 0.665 | 0.250 |

**Table S14. Paired comparisons of subjective distress within each closest relationship type between people of low and high out-degree centrality**

| Relationship type | Mean Difference (low – high) | SD | df | *p* |
| --- | --- | --- | --- | --- |
| Strangers | -0.977 | 0.561 | 48 | 0.088 |
| Familiar others | -0.156 | 0.561 | 48 | 0.783 |
| Spouse | -0.202 | 0.357 | 48 | 0.574 |

**Exploratory Analysis: Neural Sensitivity by Relationships with Partners**

We conducted additional analyses exploring the relationship between neural sensitivity to exclusion and out-degree centrality when playing with different Cyberball partners (spouse, familiar others, or stranger). To do so, we ran analogous linear models as before, predicting subjective emotional distress during social exclusion from the interaction of brain activity in each ROI and out-degree centrality, on data partitioned based on the nature of participants’ relationships with the other players.

Our results indicate that participants with high out-degree centrality showed particularly pronounced neural sensitivity to being socially excluded by strangers in the social pain and negative affect ROIs (Figure S4c, S4f), but not in the mentalizing ROI (Figure S4j). Furthermore, participants with high out-degree centrality also showed pronounced neural sensitivity to being socially excluded in all three ROIs when playing with their spouse (Figures S4a, S4d, S4g), although the magnitude of effects are much smaller compared to when playing with strangers. Overall, the results of these exploratory analyses mirror the behavioral results presented in the main text; however, we encourage caution when drawing inferences from these findings, given that the partitioned data result in smaller sample sizes than are generally recommended for examining brain-behavior associations.

**
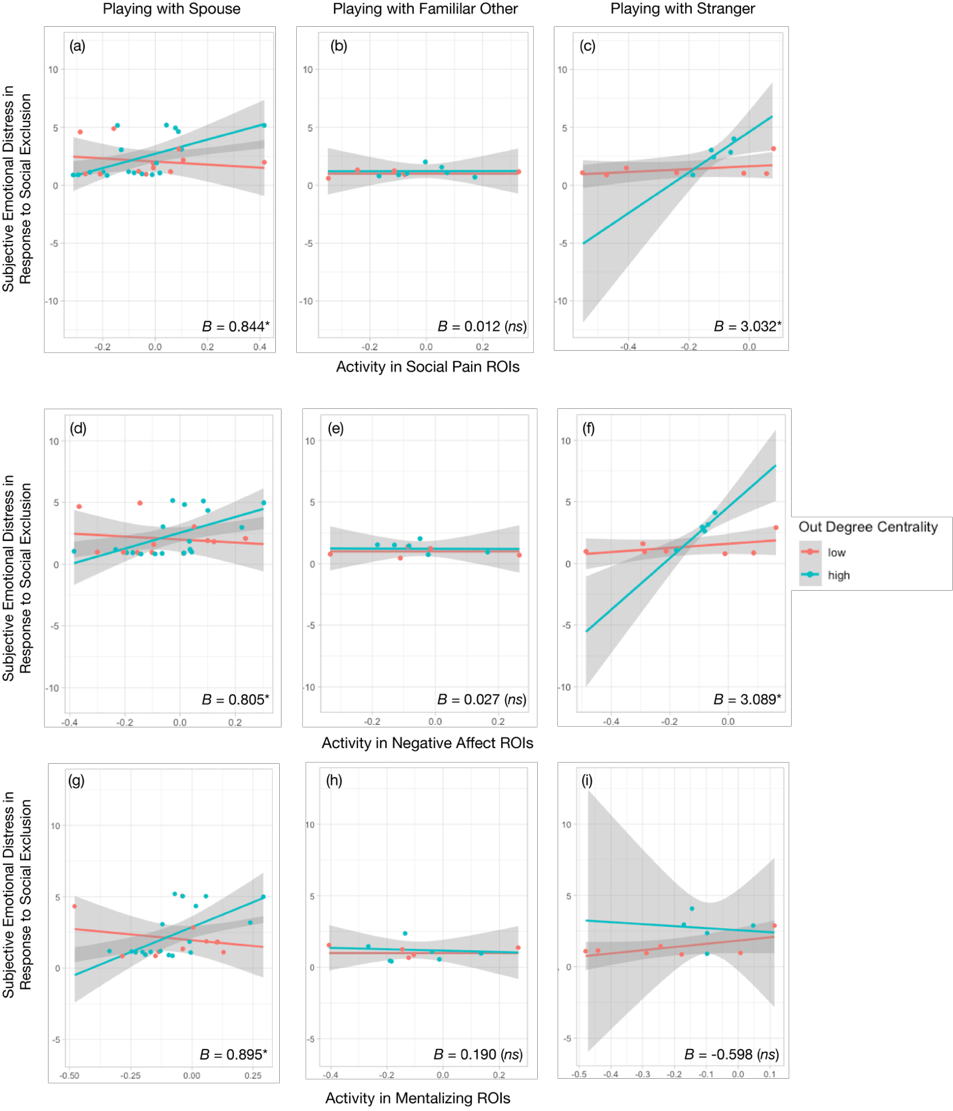
**

**Figure S4**. **Predicting subjective emotional distress during exclusion from out-degree centrality x brain activity by participants’ relationships with other players**. Corroborating the behavioral results, individuals with high out-degree centrality showed particularly greater neural sensitivity in the social pain and negative affect ROIs to social exclusion when playing with strangers (c, f). We did not observe this pattern in the mentalizing ROI (j). *B* indicates the standardized regression coefficient for the interaction term (ROI activity * out-degree centrality predicting subjective emotional distress during exclusion); **p* < 0.05, *ns* = not statistically significant with a *p* > 0.05.

**Neural Correlates of Subjective Emotional Distress – Whole-Brain Results**

To complement our *a priori* ROI results, we also conducted a whole-brain analysis to examine whether activity in regions outside of our three sets of ROIs scaled with the extent to which participants felt subjective emotional distress at being excluded. To do so, we first computed first-level models for each participant for the exclusion > inclusion contrast. Next, we fit a second-level model to relate the contrast values and participants’ subjective emotional distress using Nistats. The results of this whole-brain analysis are reported in Figure S5 at a corrected *p*-threshold of *p* < .0001 and corroborate our main results.


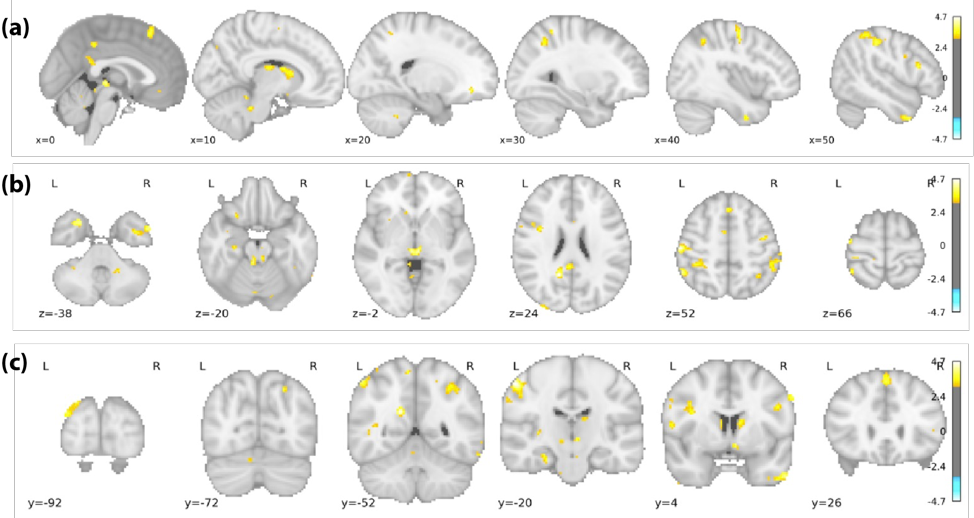


**Figure S5. Whole-brain correlates of subjective emotional distress.** Significant activations associated with subjective emotional distress across the whole-brain are visualized in (a) sagittal, (b) axial, and (c) coronal cuts of the brain (*p* < 0.001, controlling for false positive rate; minimum cluster size = 10 voxels). Our whole-brain results suggest that subjective emotional distress during social exclusion is relatively localized to our ROIs. Our whole-brain results identified regions in the mentalizing ROIs to be associated with subjective emotional distress, including regions of the temporoparietal junction, temporal pole, and the posterior cingulate cortex, as well as regions in the social pain ROIs, including the dorsal anterior cingulate cortex.

Next, to complement our ROI results showing that the relationships between ROI activity and subjective emotional distress were driven by individuals with high out-degree, we conducted whole-brain analyses to examine whether activity in regions outside of our three sets of ROIs showed similar results. To do so, we first subset our data to test the relationship between brain activity and subjective emotional distress in high and low out-degree centrality participants separately. We then fit two second-level models, one for each subset data, to relate the exclusion > inclusion contrast values and participants’ subjective emotional distress using Nistats. The results of this whole-brain analysis are reported in Figure S6 at a corrected *p*-threshold of *p* < .0001. These results corroborate our ROI results, indicating that the relationships between neural activations and subjective emotional distress were driven by the high out-degree centrality participants.


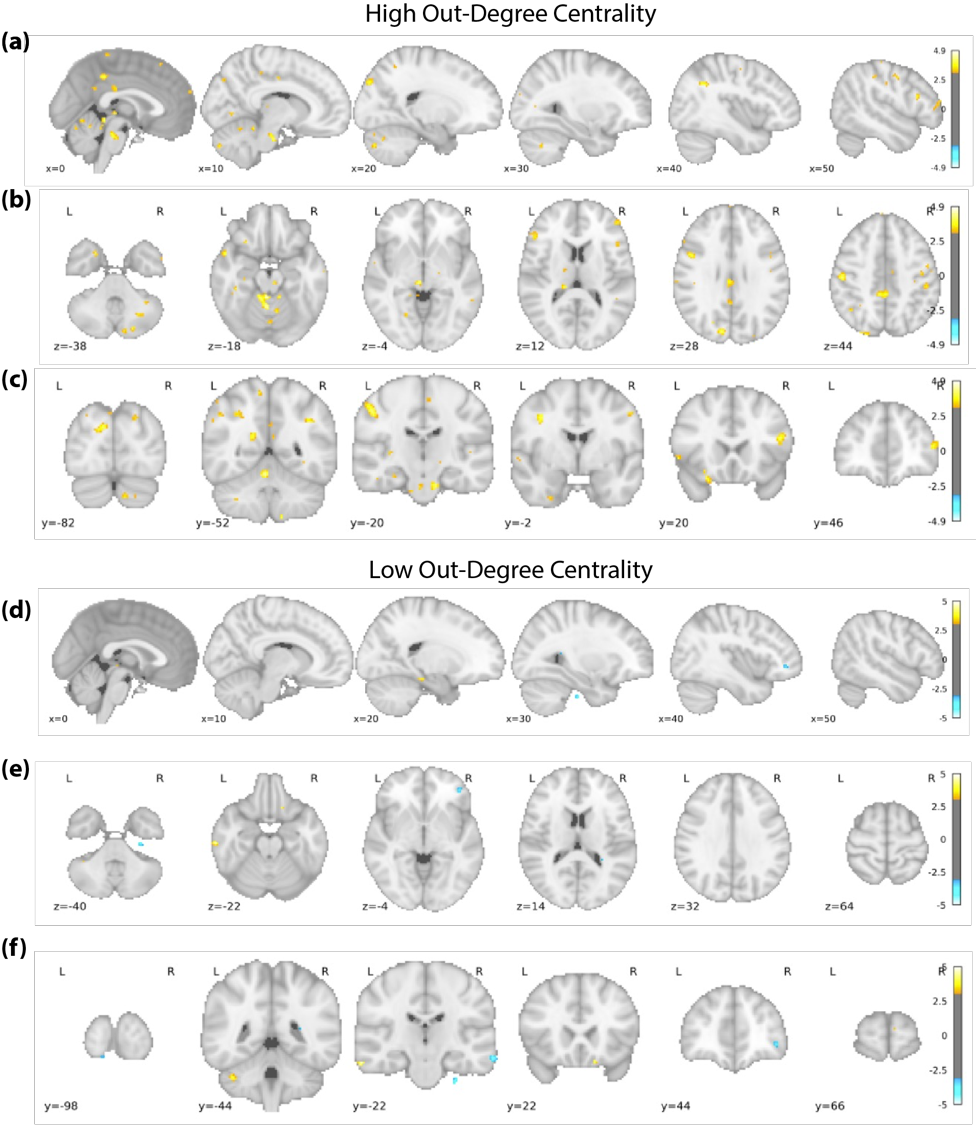


**Figure S6. Whole-brain correlates of subjective emotional distress is driven by participants with high out-degree centrality.** Significant activations associated with subjective emotional distress across the whole-brain are visualized in high (a-c) and low (d-f) out-degree centrality participants (*p* < 0.001, controlling for false positive rate; minimum cluster size = 10 voxels). Our whole-brain results corroborate our main ROI results, suggesting that the neural correlates of subjective distress during exclusion are driven by high out-degree participants.

**Using the least intimate relationship type as the relationship variable**

At the request of a reviewer, we ran the analyses using the least intimate relationship type as the relationship variable (e.g., playing with a stranger and a spouse/familiar other will be coded as playing with a stranger), such that 23 participants played with familiar others and 31 participants played with strangers. Within each group categorized by the least intimate relationship type, we did not see a significant difference in subjective feeling of distress during exclusion between people of high out-degree centrality versus people of low out-degree centrality (familiar other: *t*(21) = -0.378, *p* = 0.140; stranger: *t*(29) = 0.865, *p* = 0.394).

**Exploratory Analyses: In-degree centrality and eigenvector centrality**

At the request of a reviewer and to further leverage our sociocentric network data, we conducted exploratory analyses investigating two other centrality measures (i.e., in-degree and eigenvector centrality) and their relationships with subjective distress during social exclusion using the same methods as reported in the manuscript for out-degree centrality measures.

To assess the neural correlates of subjective emotional distress and in-degree centrality, we ran linear models predicting subjective emotional distress using the brain activity in each of the three sets of ROIs, in-degree centrality, and their interaction. The results revealed no significant interaction effects between in-degree centrality and brain activity in any set of the ROIs when predicting subjective emotional distress (Tables S13-S15). Mirroring the behavioral analyses conducted for out-degree centrality reported in the main manuscript, we also conducted pooled *t*-tests to see if people of high versus low in-degree centrality differed in their self-reported level of distress during exclusion and when playing Cyberball with different partners. These behavioral analyses yielded no significant results, such that there was no significant difference found between people of high and low in-degree centrality in terms of how distressed they felt during exclusion in general (*M*_high_ = 2.132, SD_high_ = 1.606; *M*_low_ = 1.769, SD_low_ = 1.063; *t*(56) = 1.025, *p* = 0.310) and when playing with different types of partners: strongest relationship type is spouse (*M*_high_ = 2.000, SD_high_ = 1.713; *M*_low_ = 1.667, SD_low_ = 1.029; *t*(28) = 0.662, *p* = 0.513), familiar other (*M*_high_ = 2.200, SD_high_ = 1.789; *M*_low_ = 1.857, SD_low_ = 1.215; *t*(10) = 0.398, *p* = 0.699), or strangers (*M*_high_ = 2.625, SD_high_ = 1.601; *M*_low_ = 1.813, SD_low_ = 1.193; *t*(10) = 0.998, *p* = 0.342).

We also ran analogous neural and behavioral analyses for eigenvector centrality. We found no significant interaction effects between eigenvector centrality and brain activity in any of our three ROIs (Tables S16-S18), and we found no significant difference between people of high and low eigenvector centrality in their subjective emotional distress overall (*M*_high_ = 2.040, SD_high_ = 1.541; *M*_low_ = 2.021, SD_low_ = 1.264; *t*(47) = 0.048, *p* = 0.962) and when playing with different types of partners: strongest relationship type is spouse (*M*_high_ = 2.400, SD_high_ = 1.724; *M*_low_ = 2.250, SD_low_ = 1.666; *t*(19) = 0.182, *p* = 0.856), familiar other (*M*_high_ = 1.500, SD_high_ = 1.225; *M*_low_ = 1.600, SD_low_ = 1.049; *t*(14) = -0.174, *p* = 0.864), or strangers (*M*_high_ = 1.500, SD_high_ = 1.000; *M*_low_ = 2.375, SD_low_ = 1.188; *t*(10) = -1.259, *p* = 0.237).

Table S15. Predicting participants’ subjective emotional distress in response to social exclusion from mean activity in social pain ROIs, in-degree centrality, and their interaction

| Predictor | *B* | *SE* | *p* |  |
| --- | --- | --- | --- | --- |
| Intercept | -0.090 | 0.156 | 0.565 |  |
| Activity in Social Pain ROIs | 0.366 | 0.180 | 0.047* |  |
| In-Degree Centrality | 0.284 | 0.272 | 0.301 |  |
| Activity in Social Pain ROIs*In-Degree Centrality | -0.172 | 0.257 | 0.506 |  |
| **p* <.05, ***p* <.01, ****p* <.001 | | | | |

Note: In-degree centrality is an ordered categorical variable with the reference level set to low; positive values indicate a greater association between high in-degree centrality and subjective emotional distress (the dependent variable).

Table S16. Predicting participants’ subjective emotional distress in response to social exclusion from mean activity in negative affect ROIs, in-degree centrality, and their interaction

| Predictor | *B* | *SE* | *p* |  |
| --- | --- | --- | --- | --- |
| Intercept | -0.088 | 0.152 | 0.563 |  |
| Activity in Negative Affect ROIs | 0.402 | 0.168 | 0.020* |  |
| In-Degree Centrality | 0.274 | 0.266 | 0.306 |  |
| Activity in Negative Affect ROIs*In-Degree Centrality | -0.117 | 0.251 | 0.642 |  |
| **p* <.05, ***p* <.01, ****p* <.001 | | | | |

Note: In-degree centrality is an ordered categorical variable with the reference level set to low; positive values indicate a greater association between high in-degree centrality and subjective emotional distress (the dependent variable).

Table S17. Predicting participants’ subjective emotional distress in response to social exclusion from mean activity in mentalizing ROIs, in-degree centrality, and their interaction

| Predictor | *B* | *SE* | *p* |  |
| --- | --- | --- | --- | --- |
| Intercept | -0.075 | 0.151 | 0.623 |  |
| Activity in Mentalizing ROIs | 0.377 | 0.165 | 0.027* |  |
| In-Degree Centrality | 0.228 | 0.265 | 0.393 |  |
| Activity in Mentalizing ROIs*In-Degree Centrality | -0.021 | 0.249 | 0.933 |  |
| **p* <.05, ***p* <.01, ****p* <.001 | | | | |

Note: In-degree centrality is an ordered categorical variable with the reference level set to low; positive values indicate a greater association between high in-degree centrality and subjective emotional distress (the dependent variable).

Table S18. Predicting participants’ subjective emotional distress in response to social exclusion from mean activity in social pain ROIs, eigenvector centrality, and their interaction

| Predictor | *B* | *SE* | *p* |  |
| --- | --- | --- | --- | --- |
| Intercept | -0.023 | 0.202 | 0.911 |  |
| Activity in Social Pain ROIs | 0.119 | 0.251 | 0.638 |  |
| Eigenvector Centrality | 0.098 | 0.283 | 0.731 |  |
| Activity in Social Pain ROIs*Eigenvector Centrality | 0.272 | 0.306 | 0.378 |  |
| **p* <.05, ***p* <.01, ****p* <.001 | | | | |

Note: Eigenvector centrality is an ordered categorical variable with the reference level set to low; positive values indicate a greater association between high eigenvector centrality and subjective emotional distress (the dependent variable).

Table S19. Predicting participants’ subjective emotional distress in response to social exclusion from mean activity in negative affect ROIs, eigenvector centrality, and their interaction

| Predictor | *B* | *SE* | *p* |  |
| --- | --- | --- | --- | --- |
| Intercept | -0.013 | 0.195 | 0.948 |  |
| Activity in Negative Affect ROIs | 0.109 | 0.223 | 0.627 |  |
| Eigenvector Centrality | 0.059 | 0.273 | 0.830 |  |
| Activity in Negative Affect ROIs*Eigenvector Centrality | 0.369 | 0.283 | 0.199 |  |
| **p* <.05, ***p* <.01, ****p* <.001 | | | | |

Note: Eigenvector centrality is an ordered categorical variable with the reference level set to low; positive values indicate a greater association between high eigenvector centrality and subjective emotional distress (the dependent variable).

Table S20. Predicting participants’ subjective emotional distress in response to social exclusion from mean activity in mentalizing ROIs, eigenvector centrality, and their interaction

| Predictor | *B* | *SE* | *p* |  |
| --- | --- | --- | --- | --- |
| Intercept | -0.005 | 0.192 | 0.980 |  |
| Activity in Mentalizing ROIs | 0.120 | 0.225 | 0.596 |  |
| Eigenvector Centrality | 0.012 | 0.269 | 0.963 |  |
| Activity in Mentalizing ROIs*Eigenvector Centrality | 0.382 | 0.281 | 0.181 |  |
| **p* <.05, ***p* <.01, ****p* <.001 | | | | |

Note: Eigenvector centrality is an ordered categorical variable with the reference level set to low; positive values indicate a greater association between high eigenvector centrality and subjective emotional distress (the dependent variable).

1. The same method was used to stratify the full sample into high and low out-degree groups as was used in the neuroimaging sample. [↑](#footnote-ref-1)
